# Drug-Induced Stevens-Johnson Syndrome Without Skin Manifestations: A Case Report and Review of Literature

**DOI:** 10.7759/cureus.36318

**Published:** 2023-03-17

**Authors:** Seham Alsulami, Malak Aldahasi, Hazem M Aljabri, Mazin Aljabri

**Affiliations:** 1 College of Medicine, Umm Al-Qura University, Makkah, SAU; 2 Department of Dermatology, Heraa General Hospita, Makkah, SAU; 3 Collage of Medicine, Umm Al-Qura University, Makkah, SAU; 4 Department of Dermatology, Heraa General Hospital, Makkah, SAU

**Keywords:** stevens johnson syndrome, azithromycin, drug eruption, fuchs syndrome, atypical stevens johnson syndrome

## Abstract

Stevens-Johnson syndrome (SJS) typically involves a skin rash, mucositis, and conjunctivitis. Previous reports of SJS without skin manifestations affect children and are usually associated with *Mycoplasma pneumoniae* infection. We present a rare case of oral and ocular SJS without skin lesions in a healthy adult after exposure to azithromycin without mycoplasma pneumonia infection.

## Introduction

Fuchs syndrome is a rare variant of Stevens-Johnson syndrome (SJS) with an absence or a few cutaneous manifestations. The lesions are specifically found on oral, ocular, and genital mucosae [1]. It is a sporadic disease with different terms in the literature: "Stevens-Johnson syndrome without skin lesions" or "atypical Stevens-Johnson syndrome" or "incomplete Stevens-Johnson syndrome" or "Mycoplasma pneumoniae-associated mucositis" (MPAM) or "MP-induced rash and mucositis (MIRM)" [1,2].

It is commonly associated with *Mycoplasma pneumoniae* infection, and most of the cases are reported in children and adolescents but may affect adults [1,2]. Atypical SJS predominantly occurs in males (66%), with oral mucosa being mostly affected, followed by conjunctival and urogenital [1,2]. The cause is not always clear; therefore, a careful and detailed clinical examination is required to eliminate a differential diagnosis. Treatment is mainly supportive and includes discontinuing offending medications, fluids, and nutritional support. In addition, antibiotics, systemic corticosteroids, and intravenous immunoglobulins (IVIG) can be used [1]. We present a rare case of oral and ocular SJS without skin lesions in a healthy adult after exposure to azithromycin without mycoplasma pneumonia infection.

## Case presentation

A 28-year-old female patient with no prior medical history presented to the emergency department with a two-day history of painful lip erosions and difficulty swallowing. No other mucosal areas were affected, and there were no skin lesions. She was diagnosed with herpetic stomatitis and was discharged on oral acyclovir. After two days, she returned to the emergency department with poor oral intake due to painful lip erosions and a sore throat. She also had a bilateral conjunctival injection. We were consulted at that time, and upon detailed history, the patient stated that she was seen by a general practician six days back for upper respiratory tract infection symptoms, and she was given azithromycin for three days.

**Figure 1:**
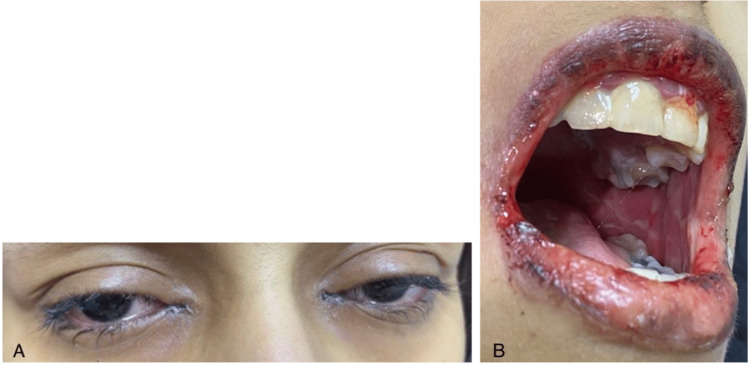
Atypical Stevens Johnson syndrome initial presentation (A) bilateral conjunctival injection, (B) mucositis and erythematous lip erosions

On the physical examination, the patient looked well and was not in distress; there were erosions on both lips and the injected conjunctiva (Figure 1) and no genital or skin lesions. She was admitted, and treatment was initiated with intravenous fluid, intravenous acyclovir 10 mg/kg every eight hours, and an oral nystatin wash. Otolaryngologists and ophthalmologists were consulted. Local treatment with ciprofloxacin eye drops, fusidic acid eye drops, and artificial tears was applied as ophthalmology recommends.

Investigations showed that the complete blood count and urinalysis were normal. Inflammatory markers C-reactive protein 10.7 mg/dl (normal <0.3 mg/dl) and erythrocyte sedimentation rate 103 mm\hr (normal 0-20) were both elevated. Serum *M. pneumoniae* antibody immunoglobulin G (IgG) and immunoglobulin M (IgM) and Herpes IgM were negative. The chest X-ray was unremarkable. The patient was offered to do a biopsy of the oral mucosa, but she refused. Both urine and sputum cultures were found to be negative.

At that time, the acyclovir was stopped, and the primary diagnosis was atypical SJS with no skin lesions. She started on intravenous hydrocortisone, 100 mg every eight hours. After four days, the patient markedly improved, tolerating oral therapy, and the lip erosions improved. She was discharged on prednisone 40 mg once daily for one week, then to be tapered by 5 mg weekly.

## Discussion

SJS is a severe mucocutaneous reaction that affects the skin and mucous membranes and includes erythema multiforme minor, SJS (erythema multiforme major), and toxic epidermal necrolysis [3]. More than 90% of patients have skin involvement; skin lesions can range in appearance from targetoid to diffuse erythema [4]. Common areas for mucosal lesions include the mouth, eyes, and genitalia. The most frequently identified cause in children is an infection, with *M. pneumoniae* being the most prevalent infectious agent linked to SJS in all age groups [3]. Adults’ most frequent precipitants are nonsteroidal anti-inflammatory drugs, allopurinol, sulfonamides, and aromatic antiepileptics [3,4].

Atypical SJS is a rare variation of SJS with a complete absence of or only a few cutaneous manifestations. It is usually seen in children infected by *M. pneumoniae* [1]. To the best of our knowledge, only a few cases of isolated mucosal SJS caused by medication have been reported without clinical or laboratory evidence of *M. pneumoniae* infection. In the first case, the patient presented with ulcerations in the lips and gums and bilateral conjunctival congestion after voltalin and sulfasalazine [5]. Ishiguro et al. reported drug-induced SJS-like eruptions predominating in mucosa following meloxicam and tizanidine [6]. Moreover, isolated mucositis can be a rare complication when combining both trimethoprim/sulfamethoxazole (TMP-SMX) and methotrexate [7]. Canter and Smith et al. [8] reported incomplete SJS caused by a single medication, TMP-SMX, without any clinical or laboratory evidence of *M. pneumoniae* infection. Anders et al. described a case of a 14-year-old male with atypical Stevens-Johnson syndrome caused by azithromycin [9].

Fuchs’ syndrome is difficult to diagnose. Therefore, a careful and detailed clinical examination is needed, and a biopsy of oral lesions should also be taken to support the diagnosis. In our case, the patient was offered to have a biopsy, but she refused.

Treatment is mainly supportive and includes discontinuing offending medications, providing fluids and nutritional support, and controlling pain. Moreover, antibiotics, systemic corticosteroids, and intravenous immunoglobulins (IVIG) can be used [1]. Our patient improved with the discontinuation of the offending antibiotic and steroids. She was informed to avoid azithromycin.

## Conclusions

Fuchs syndrome is a rare variant of SJS with an absence of or a few cutaneous manifestations. Therefore, the diagnosis needs a careful and detailed clinical examination. Supportive treatment with fluids, discontinuation of the offending drug, and long-term care with ointments and anti-inflammatory medications should be administered accordingly.
